# Does Migration Distance Affect Happiness? Evidence From Internal Migrants in China

**DOI:** 10.3389/fpubh.2022.913553

**Published:** 2022-05-30

**Authors:** Ge Zheng, Dongliang Yang, Jiawei Li

**Affiliations:** ^1^Department of Regional Economics, School of Northeast Asian, Jilin University, Changchun, China; ^2^Northeast Asian Research Center, Jilin University, Changchun, China; ^3^Department of Accounting, School of Economics and Management, Changchun University of Technology, Changchun, China

**Keywords:** migration distance, happiness, social integration, mental health, Chinese internal migrants

## Abstract

**Background:**

Happiness is a complex concept involving many subjects such as society, psychology, and ethics. How will migration distance affect migrants' happiness under the new trend of migration in China? The goal of this paper is to analyze the influence and transmission mechanism of migration distance on happiness of migrant individuals, and the heterogeneity of this effect on urban and rural migrants.

**Methods:**

Employing data of 129,803 observations from the 2012 China Migrants Dynamic Survey, we first estimate the effects of migration distance on happiness by the ordinal logistic regression and propensity score matching (PSM) method. Second, we examine the heterogeneity of effect by splitting the sample into the urban and rural migrants. Finally, we analyze the transmission mechanism of migration distance on happiness by mediating effect model.

**Results:**

The migration distance of internal migrants in China has a significant negative impact on happiness. Urban migrant individuals show a stronger response to migration distance compared to rural counterparts. Social integration is proved as the potential mechanism through which the effect of migration distance on happiness.

**Conclusion:**

The results emphasize happiness of internal migrant and other mental health problems. Moreover, particular attention should be paid to social integration on happiness, such as strengthening the cultural exchange in different areas, narrowing the income gap between urban and rural areas, promoting rational migrant decision of individual, and enhancing the happiness of them.

## Introduction

Happiness is a complex concept involving many subjects, such as society, psychology, and ethics. For example, happiness is defined as positive and pleasurable emotions that come from heart (1); happiness is described as a beautiful experience of reaching a higher level of life by exerting one's potential (2); or emphasizing an individual's rational cognition of happiness, and judging happiness from the comparison between the preset happiness standard and the real life situation (3). In recent years, the pursuit of happiness in society has continued to heat up. Happiness is not only a comprehensive reflection of individual mental and physical health but also a universal goal and expectation in human life all over the world. To this end, the 66th United Nations General Assembly proclaimed March 20 as the International Day of Happiness in 2012. The resolution is of practical importance to the well-being of all people.

With the rapid development of economy and society of China, internal migration of individuals presents a proximity trend. According to the data of China's seventh population census, the migrant population of China was 375,816,800 in 2020, accounting for 26.63% of the total population, among which the intra-province migrant population was 250,979,600, and the inter-provincial migrant population was 124.84 million, increasing respectively by 84.5 and 45.3% compared with 2010 (4). About two-third of the migrant population belongs to the intra-province migrant population.

Suppose high welfare is the ultimate goal of all human efforts (5). The purpose of migrant individual is to pursue higher happiness. In that case, it seems easy to explain why more and more Chinese migrants choose to move at short distances. Short-distance migration reflects the preference of destination of the migrants, as well as the spiritual belonging and social welfare that the destination can provide to the migrants (6). Fundamentally, it is likely that people choose to migrate in proximity out of comparison and acquisition of happiness. Therefore, one problem arises naturally, which is does migration distance affect happiness?

In addition to the length of geographic distance, migration distance represents the psychological distance between the individuals and their relatives in the hometown. This psychological distance includes language differences, climate differences, dietary customs, living habits, cultural education, etc. These are regional cultures with local characteristics formed over a long historical period (7). Regional cultures are mainly distributed across China in the form of provincial administrative regions (8). In the same province, people are influenced by physical geography and cultural environment. They share similarities in character, behavior, habit, thinking, and even personality. These factors significantly pull the psychological distance between people. The provincial-level proximity in China is conducive to people's rational adjustment to social environment or life pressure, forming good mental health, and thus maintaining a high sense of happiness (9–11).

On the one hand, under the influence of Confucian culture, Chinese society paid more attention to clan and family relationships. In the traditional concept of elderly support, “While parents are still living, it is better for child not to stay far away from home” and “Raise children to care for you when you get old.” “Serving on the side” and other support modes are regarded as the criteria for filial piety to parents, which restricts the range of migration to a certain extent, leading people to choose short-distance migration.

On the other hand, in long-distance migration, the difference in cultural environment is an inevitable problem for the migrant population. Previous studies have found that the farther the dialect distance is, the weaker the migrant population's sense of identity and trust in the city, and the weaker their willingness to settle down and happiness (12, 13). Moreover, the sense of happiness reflects personal, economic, and spiritual levels, and the migrant population needs to make corresponding cognitive and emotional evaluations when the living environment changes and forms a subjective psychological response (14). The long-distance migration process lacks the support and care of relatives and friends, which can easily lead to the disintegration of the family and the loss of traditional values, reduce the individual's psychological sense of belonging and social integration willingness to the destination, and happiness naturally declines (15, 16). Based on the above analysis, we propose Hypothesis 1.

Hypothesis 1. Migration distance has a significant negative effect on happiness.

China's population distribution presents a clear urban-rural dual structure. Due to China's preference for urban policies and resources, there are considerable differences in social welfare and public security between urban and rural population based on the household registration system. The income level of urban residents has long been at an advantage compared to the rural ones. According to the “happiness paradox” proposed by Easterlin (17), income and happiness have no apparent positive correlation. Some existing empirical studies showed that individuals with high-income levels were more likely to face disappointment in income expectations and the decline in happiness caused by the disappointment of expectations (18–22). Hence, Hypothesis 2 arises.

Hypothesis 2. Urban individuals show a stronger response to migration distance compared to rural counterparts.

For some long-distance migrants, the unhappiness brought by the difficulty of integrating into the place of migration may be more prominent (23). Due to the persistent forms of discrimination that migrants experience, which leads to the marginalization and exclusion of migrant individuals, they continue to fall victims of being left behind. For example, in allocating public resources such as medical education and other public resources, the local residents and the foreign residents are treated differently. Zheng (24) found that migrating to a new region creates a sense of alienation and stress, which puts the migrating individuals at an extremely high risk of mental illnesses such as depressive symptoms. Happiness is a complete manifestation of mental health. If migrants have a good mental health state, such as optimism and positivity, they will remain happy. In contrast, if mental health problems such as worry and distress increase, it will undoubtedly reduce the experience of happiness (25). Hence, Hypothesis 3 is proposed.

Hypothesis 3. Migration distance can harm happiness through their effects on social integration.

The novelty of this paper is four-fold. First, to the best of our knowledge, this is the first paper to investigate the impact of migration distance on happiness for the case of a developing country: China. Previous studies focused on the impact of education and income on happiness (24, 26). Our research investigates the general impact of migration distance as an external shock on happiness. Second, we address the impact by propensity score matching (PSM) method, which is important to identify the casual effect of migration distance on happiness. Third, compared with empirical research in the existing literature, we examine the heterogeneity of effects by splitting the sample into urban and rural areas. These results help us understand why urban individuals showing a stronger response to migration distance than rural counterparts. Finally, this paper reveals that migration distance could affect happiness through the influence of social integration. The causal effect of migration distance on happiness is verified.

In this paper, we examine the effect of migration distance on happiness. Employing 129,803 research individuals in the 2012 China Migrants Dynamic Survey, we test Hypotheses 1–3, respectively and found that migration distance has a significant negative impact on happiness. This negative impact is remarkably different between urban and rural migrant individuals. In addition, social integration is proved to be the potential mechanism through which the effect of migration distance on happiness.

## Data and Variables

### Data

The data used in this paper are mainly from the 2012 China Migrants Dynamic Survey (CMDS), which was organized and implemented by the Migrant Population Service Center of the National Health Commission of China (formerly the National Population and Family Planning Commission). This data is the only national survey data on happiness released by government departments. Compared with other survey data in China, it is highly credible and authoritative. The data is based on the 2011 annual report data of all the migrant individuals of 31 provinces as the basic sampling frame and adopts the stratified, multi-stage, and scale-proportional PPS method for sampling. The survey respondents lived in the immigration area for more than 1 month and were 15–59 years old migrant individuals with no household registration in the district (county, city). The total sample size of the national survey is 159,000 people. The survey content involves information such as the migration direction, social integration, and happiness of the migrant individuals. According to the research purpose of the article, we eliminate outliers and invalid values in the sample and finally obtain 129,803 observations.

### Variable Definitions

The dependent variable is happiness. As a subjective feeling of an individual, happiness is objectively immeasurable. For this problem, discrete numerical variables can well show individual wishes and be compared and analyzed (27). In the questionnaire: “Compared with your hometown (where you migration from), do you feel happy now?” The respondents' options include very happy, happy, so-so, unhappy, and very unhappy, and the corresponding values are as follows: 5, 4, 3, 2, and 1, with higher numbers indicating greater happiness. The number of people who answered very happy, happy, so-so, unhappy, and very unhappy in the sample was respectively 18,535 (14.28%), 61,637 (47.49%), 47,460 (36.56%), 1,930 (1.49%), 241 (0.19%).

The independent variable is migration distance. The migration distance refers to the range of individual's movement, which is the main factor for the migrant individuals to make migrant decisions (28). The information on the migration distance in the questionnaire is in the individual basic situation module, and the latest migration range is judged based on the household registration and current residence of the migrant individual. Dummy variables capture migration distance. If the migrant individual chooses Inter-provincial migration, it is 1; otherwise, it is 0. In the sample data, the number of people who chose intra-provincial and inter-provincial migration were 55,176 and 74,627, and the corresponding proportions were about 42.51 and 57.49%, respectively.

The mediating variable is the willingness to integrate into the destination. Intention to integration has been identified as a potential mediator between migration distance and happiness. Immigrants need to face spiritual costs such as being far away from home, family, relatives, and friends. Research suggests that the lower the spiritual cost, the higher the probability of migration. These unmeasured costs affect the decision-making of the labor force at a deeper level. More importantly, the social integration process of the migrant individuals in the area is also a psychological process of adapting to the new environment, overcoming conceptual differences, and narrowing social exclusion, which directly affects a person's sense of belonging and mental health. Mentally healthy individuals are more able to feel the existence and strength of happiness. In order to objectively measure the degree of social integration of the migrant individuals, we use the question, “I think the locals are willing to accept me as one of them,” in the questionnaire to represent the degree of social integration of the migrant individuals. The corresponding answers options include completely disagree, disagree, basically agree, and fully agree. We divide it into two categories: agree and disagree.

Control variables are mainly reflected in individual heterogeneity. In China, where diplomas are the criteria for talent selection, for this reason, happiness is closely related to education. Education changes the cognitive abilities of individuals, and people with higher education are happier (29). We divide educational attainment into lower and higher education. Employment status and age may have an impact on the level of individual happiness and have 15 to 20% explanatory power for individual differences in happiness (30). Employment status refers to employees, employers, and self-employed workers. An increase in personal relative income significantly impacts happiness (31–34). Research has found that migrants can derive happiness from increased economic income and seem happier than those who stay in their country of origin (35, 36). We choose the logarithm of monthly income to represent the income level of the migrant individuals in the questionnaire, “how much was your income last month (or last employment).” In addition, Gender differences between men and women may also impact happiness (37). Research suggests that the more children need to raise, the more passion and energy need to live, and the less happiness.

### Descriptive Statistics

Table 1 describes the overall sample and generalized characteristics. It shows that the average happiness score of migrant individuals of China is 3.742, indicating that the happiness of them is generally high. From the age distribution of the migrant individuals, the average age is 34.019, indicating that the migrant individuals are primarily young people. The average time of the migrants is 4.423 years, indicating that most migrant individuals focus on medium and long-term migration rather than short-term migration. The above conclusions are only preliminary judgments and have not been obtained through rigorous hypothesis testing. However, these results reveal some interesting differences in the happiness characteristics of heterogeneous and mobile individuals.

**Table 1:** Descriptive statistics of the key variables.

**Variables**	**Definitions**	**Mean**	**SD**	**Min**	**Max**
Happiness	1 = very unhappy, 2 = unhappy, 3 = so-so, 4 = happy, 5 = very happy	3.742	0.720	1	5
Migration distance	0 = Intra-province, 1 = Inter-province	0.575	0.494	0	1
Gender	1 = female, 0 = male	0.408	0.492	0	1
Age	Respondent's age	34.019	8.914	18	59
Household register	0 = rural, 1 = urban	0.159	0.365	0	1
Income	Individual's income (in log)	7.856	0.574	4.605	11.493
Education level	0 = low, 2 = high	0.315	0.464	0	1
Employment status	0 = employees,1 = employers,2 = self-employed	0.721	0.904	0	2
Number of children	Number of children owned by the respondent	1.379	0.715	0	7
Migration time	The length of the last visit to this city/district/county	4.423	4.637	0	51
Social integration	0 = Disagree, 1 = Agree	0.929	0.256	0	1

## Models and Methods

First, we use ordinal logistic regression to empirically analyze migration distance's effect on happiness. Ordinal logistic regression is a standard method for dealing with ordinal categorical dependent variables, and it is widely used in questionnaire research. According to the level of happiness questionnaire, the dependent variables are set to 1, 2, 3, 4, and 5, representing very unhappy, unhappy, so-so, happy, and very happy. The corresponding probabilities are defined as π_1_, π_2_, π_3_ ,π_4_ and π_5_. We construct the cumulative logistic regression model as follows:


(1) $$\begin{array}{rcl} logit\frac{\pi_{1}}{1-\pi_{1}} & = & -\alpha_{1}+\beta_{1}x_{1}+\ldots +\beta_{p}x_{p} \end{array}$$

(2) $$\begin{array}{rcl} logit\frac{\pi_{1}+\pi_{2}}{1-\left(\pi_{1}+\pi_{2}\right)} & = & -\alpha_{1}+\beta_{1}x_{1}+\ldots +\beta_{p}x_{p} \end{array}$$

(3) $$\begin{array}{rcl} logit\frac{\pi_{1}+\pi_{2}+\pi_{3}}{1-\left(\pi_{1}+\pi_{2}+\pi_{3}\right)} & = & -\alpha_{1}+\beta_{1}x_{1}+\ldots +\beta_{p}x_{p}\; \end{array}$$

(4) $$\begin{array}{rcl} logit\frac{\pi_{1}+\pi_{2}+\pi_{3}+\pi_{4}}{1-\left(\pi_{1}+\pi_{2}+\pi_{3}+\pi_{4}\right)} & = & -\alpha_{1}+\beta_{1}x_{1}+\ldots +\beta_{p}x_{p} \end{array}$$

In contrast to binary logistic regression analysis, π_1_,π_1_+π_2_,π_1_+π_2_+π_3_,π_1_+π_2_+π_3_+π_4_ refer to the cumulative probability of orderly value levels of response variables. We assume that the coefficients of the respective variables remain constant, and the constant terms are variable.

Second, we use the propensity score matching (PSM) technique to address endogeneity problems. The migrants sample used in this case may have a problem of selection bias. Shamsuddin (28) accepts that happiness affect the individual's migration distance and expected staying time; people who live happily are often happy in the place they migrated. The core idea of PSM is to find the control group individuals (intra-provincial migrant individuals) with similar characteristics to the treatment group (inter-provincial migrant individuals) according to the propensity score. Using the observed results of the intra-provincial migrant individuals to estimate potential outcomes unobserved by the inter-provincial migrant individuals, the causal effect of migration distance on happiness was identified by comparing the observed and estimated results of the inter-provincial migrant individuals (38, 39). An essential assumption of PSM is that the experiment is completely randomized, and the randomized assignment of interventions does not introduce confounding bias.

Average treatment effect of the treated (ATT) refers to the average causal effect of treated (*D*_*i*_ = 1) on individuals in the treatment group, and its formula is:


(5) $$\begin{array}{r} \tau_{ATT}=E\left[Y_{1i}-Y_{0i}|D_{i}=1\right] \end{array}$$

**Figure 1:**
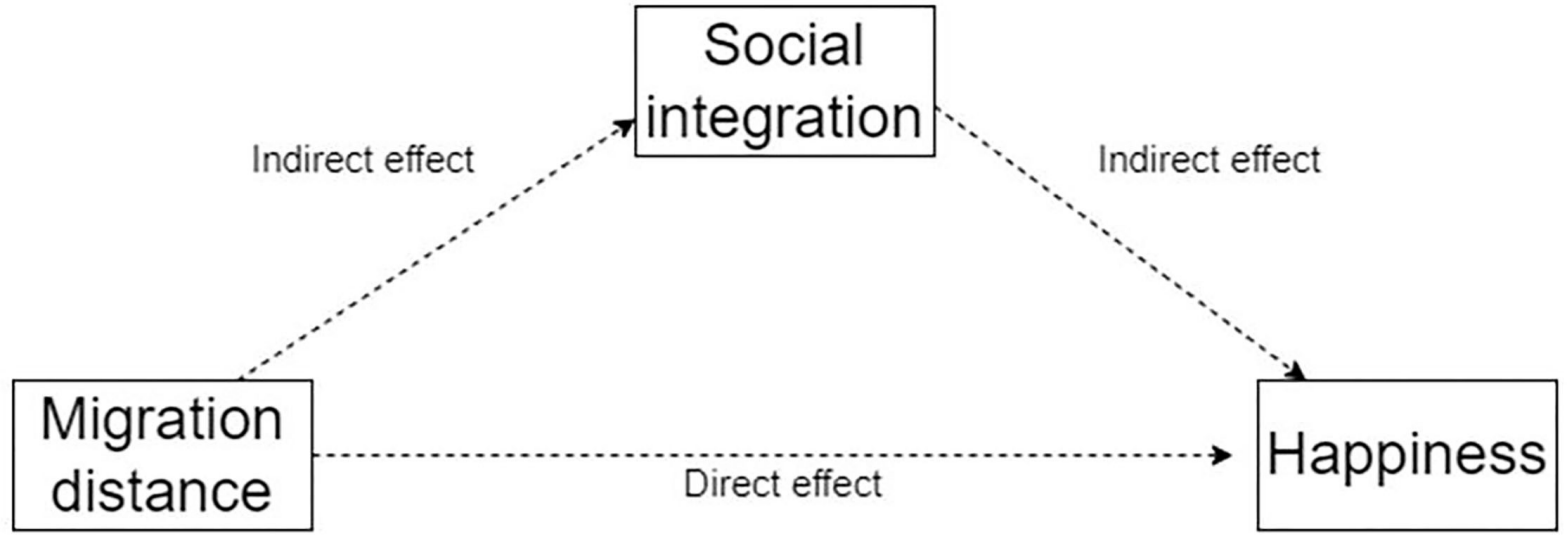
Framework of mediation effect analysis.

Third, we drive a mediation effect test based on the mediating effect model (40, 41), analyze the conduction path of the influence of migration distance on happiness, and use social integration variables to analyze the mediation effect. The analysis framework is shown in Figure 1.

## Results

### Baseline Regression Results

First of all, we use ordinal logistic regression to analyze the causal relationship between migration distance and happiness in Table 2. Column 1 controls the relevant influencing factors such as gender, age, income, education, and migration duration of the migrant individuals. The estimation results show that without considering other influencing factors, the migration distance has a significant negative impact on happiness, which is −0.367.

**Table 2:** The effect of migration distance on happiness.

**Variables**	**Happiness**	**Margin effects**				
		**Very unhappy**	**Unhappy**	**So-so**	**Happy**	**Very happy**
Range	−0.367***	0.001***	0.005***	0.077***	−0.036***	−0.047***
	(−29.80)	(11.50)	(23.30)	(30.16)	(−29.24)	(−29.46)
Gender	0.113***	−0.000***	−0.001***	−0.024***	0.011***	0.014***
	(8.98)	(−7.28)	(−8.73)	(−8.99)	(8.96)	(8.97)
Age	0.004***	−0.000***	−0.000***	−0.001***	0.000***	0.001***
	(4.89)	(−4.56)	(−4.85)	(−4.90)	(4.89)	(4.89)
Household register	0.083***	−0.000***	−0.001***	−0.018***	0.008***	0.011***
	(4.54)	(−4.26)	(−4.50)	(−4.54)	(4.53)	(4.53)
Education	0.070***	−0.000***	−0.001***	−0.015***	0.007***	0.009***
	(4.59)	(−4.31)	(−4.56)	(−4.60)	(4.59)	(4.59)
Income	0.157***	−0.000***	−0.002***	−0.033***	0.015***	0.020***
	(14.48)	(−9.45)	(−13.50)	(−14.52)	(14.40)	(14.45)
Employers	0.187***	−0.000***	−0.002***	−0.039***	0.017***	0.024***
	(9.62)	(−7.89)	(−9.82)	(−9.76)	(10.48)	(9.27)
Self-employed workers	0.102***	−0.000***	−0.001***	−0.021***	0.010***	0.013***
	(7.65)	(−6.57)	(−7.57)	(−7.66)	(7.70)	(7.61)
Children	−0.029***	0.000***	0.000***	0.006***	−0.003***	−0.004***
	(−3.08)	(2.99)	(3.07)	(3.08)	(−3.08)	(−3.08)
Time	0.036***	−0.000***	−0.000***	−0.008***	0.004***	0.005***
	(28.03)	(−11.39)	(−22.44)	(−28.29)	(27.41)	(27.81)
/cut 1	−0.346***					
	(−3.71)					
/cut 2	1.295***					
	(13.86)					
/cut 3	1.395***					
	(14.93)					
/cut 4	1.407***					
	(15.06)					
Observations	101,409	101,409	101,409	101,409	101,409	101,409

t statistics in parentheses, ***p < 0.01.

To better understand the relationship between migration distance and happiness, we calculated the mean marginal effect of each explanatory variable to reveal that the unit change of the explanatory variable affects the probability of happiness taking each value. The estimation results in columns 2 to 6 show that when all explanatory variables are at the mean value. Compared with the intra-province migrant individuals, the probability of feeling very unhappy, unhappy, so-so, happy, and very happy will change by 0.001, 0.005, 0.077, −0.036 and −0.047 respectively at the significant level of 1% for the inter-province migrant individuals outside the province when the migration distance increases by one level. The probability of individuals feeling very happy is 0.011 lower than the probability of happiness. The results show that the farther the migrant individuals move, the lower the probability of happiness. That reflects that migration distance has a negative impact on happiness. Long-distance migration is not conducive to the happiness of the migrant individuals. On the contrary, short-distance migration is conducive to people's pursuit of happiness.

In addition, from other explanatory variables, the probability of an individual feeling very happy is 0.002 higher than the probability of happiness for each additional level of higher education compared with low education. When the individual's income is higher, the probability of obtaining happiness is higher. The happiness level of women is significantly higher than that of men. The happiness level of the non-agricultural migrants is significantly higher than that of agriculture. The probability, that an individual feels very happy, decreases by 0.004 with a new baby.

### Endogeneity

In order to solve the estimation error of estimation results that may be caused by labor characteristics and selection bias, we consider the use of PSM to identify the causal relationship between migration distance and happiness. In this case, happiness is taken as the outcome variable, intra-provincial migrants are taken as the control group, and inter-provincial migrants are taken as the treatment group.

**Figure 2:**
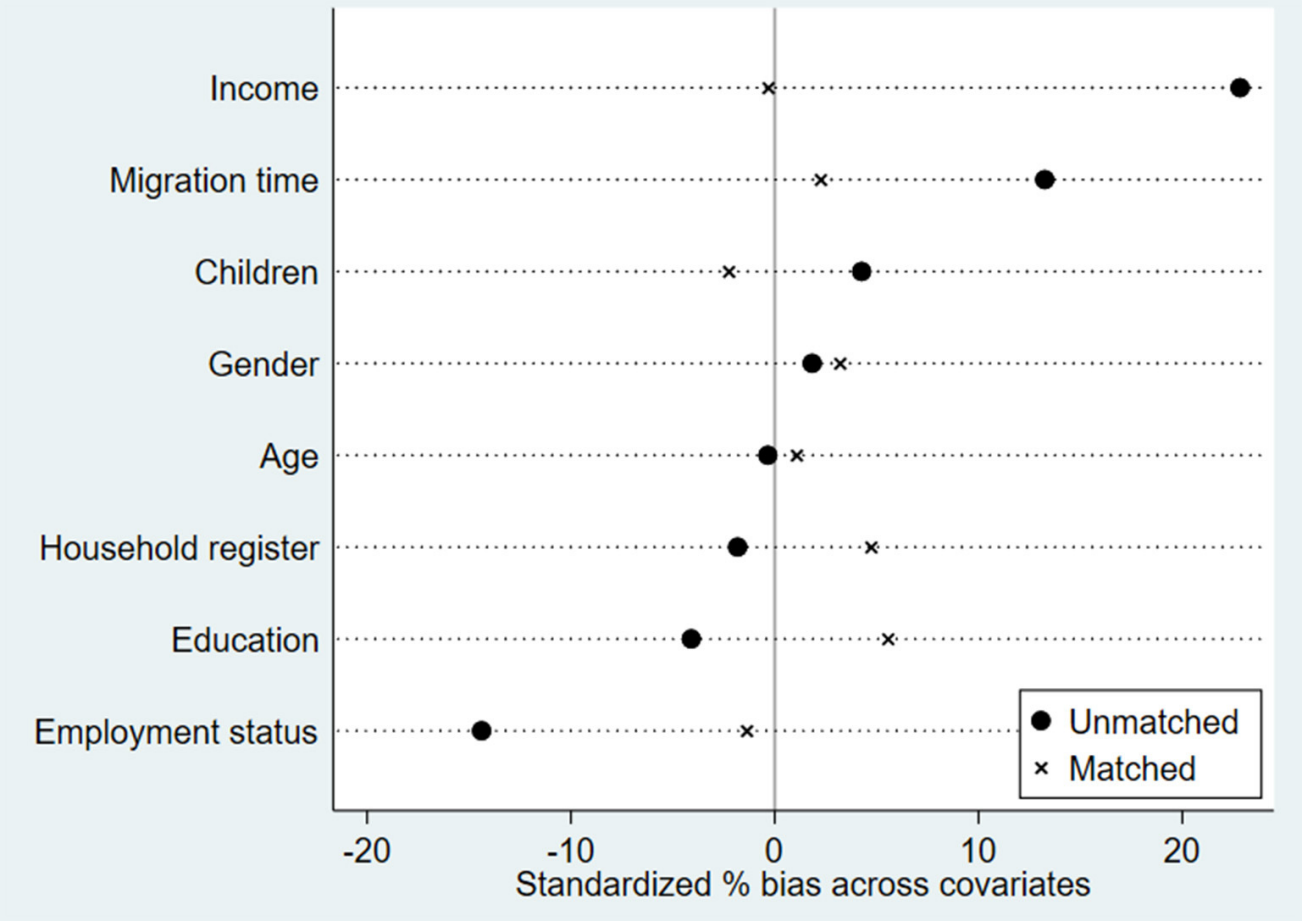
Standardized bias before and after matching.

The condition for PSM requires that the covariates of the treatment and control groups are similar, meaning that the treatment and control groups have similar constructive characteristics. If the deviation of the covariates between the two groups is too large, the PSM cannot be directly used for estimation. Therefore, we need to test the balance of the covariates first (Figure 2). After matching, the *t*-test of covariates such as gender, number of children, and time in the treatment group and the control group rejected the null hypothesis of no systematic difference between the treatment group and the control group, indicating that some covariates were poorly balanced. Considering that the standardized mean difference of all covariates decreased significantly, the standardized deviation of most variables is < 5%, which meets the requirement of a 10% balance, indicating the characteristics of the treatment group and the control group is very similar. The balance of the covariates could meet the requirements of randomized experiments.

**Figure 3:**
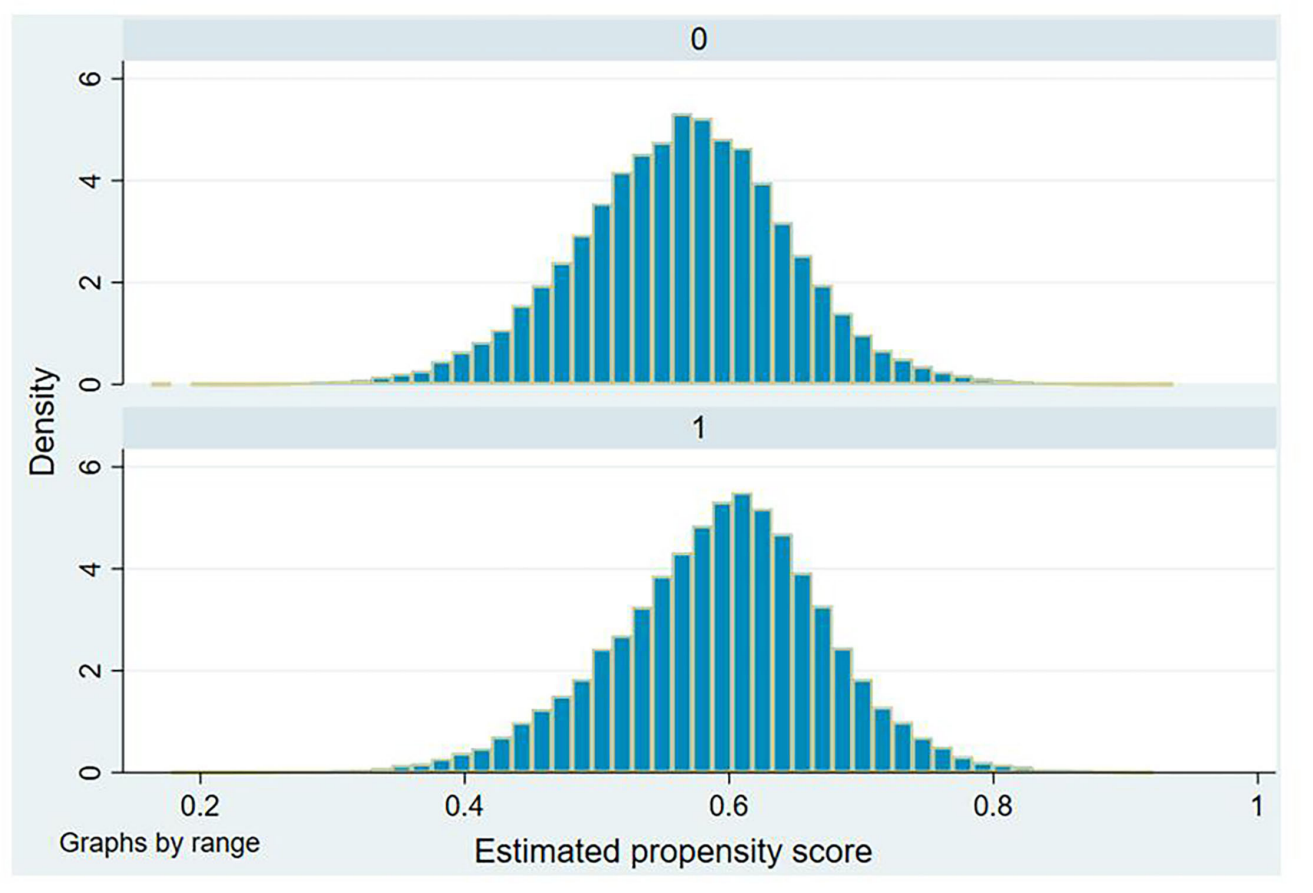
Density distribution of the propensity score.

Another essential condition for implementing matching is to meet the common support assumption: the propensity score, *pϵ*(0, 1). Therefore, when PSM is completed, we draw a histogram of the propensity score distribution of the treatment group and the control group to judge whether the results of the two groups meet the requirements of common support (Figure 3). The differences in the distribution of individual propensity scores between the control group and the treatment group are observed. The result shows that distributions of the two groups are similar, indicating that the covariates pass the balance test.

This paper presents different types of matching estimators to accurately identify the causal relationship between migration distance and happiness, including nearest neighbor matching (*k* = 1, *k* = 4), kernel matching, local linear regression matching, and mahalanobis matching. It is worth noting that the K-nearest neighbor matching method involves the selection of *k*, which is to determine the number of individuals most similar to the matching object, and nearest neighbor matching (*k* = 4) can obtain minimal mean square error (42). Table 3 shows the estimated results of different matching strategies. ATT is significantly negative, indicating that the migration distance is negatively related to happiness.

**Table 3:** PSM analysis of the effects of migration distance on happiness.

**ATT**	**Nearest Neighbor Matching (*k* = 1)**	**Nearest** **Neighbor** **Matching (*k* = 4)**	**Kernel matching**	**Local linear regression matching**	**Mahalanobis matching**
Difference	−0.131***	−0.136***	−0.137***	−0.143***	−0.135***
S.E.	0.006	0.005	0.005	0.006	0.005
T-stat	−21.47	−26.08	−29.47	−23.31	−25.95

***p < 0.01.

Combined with the characteristics of the data in this paper, we use the pseudo treatment method to test for independence. The basic idea is to use the variables of the control group as pseudo-results to test and control covariates such as age, gender, education, and employment status of the migrants, and then use the pseudo-results to estimate whether the causal effect is zero. In particular, the original control group (intra-province migrant individuals) is randomly divided into two groups: one group is selected as the treatment group and the other group as the control group.

According to the nearest-neighbor matching (*k* = 4) results in Table 4, the estimated *T*-test result of ATT was 0.43, which failed the 1% significance test, indicating that there was no significant difference in the happiness of the migrant individuals in the “treatment group” and the “control group” of the pseudo-intervention. The pseudo treatment test can not completely confirm that the independence assumption is valid, but at least it does not show that the independence assumption is invalid, which indicates that the estimation result of nearest neighbor matching has certain credibility. The results verify Hypothesis 1.

**Table 4:** Conditional independence assumptions test results for pseudo treatment.

**Nearest neighbor matching**	**Difference**	**S.E**.	**T-stat**
*k* = 1	−0.002	0.009	−0.2
*k* = 4	0.003	0.008	0.43

*For data with large samples and normal distribution, when 1.65 < |t| <1.96, p <0. 10; when 1.96 < |t| <2.58, p <0.05; when |t|>2.58, p < 0.01*.

### Analysis of Urban and Rural Heterogeneity

To better understand the causal effect of migration distance on happiness, we divide the sample according to the migrant individual's household registration characteristics and gender characteristics and then estimate the impact of heterogeneity.

Table 5 shows the heterogeneous effects of migration distance on happiness for rural and urban migrant individuals. The key variable coefficients of the equation all pass the 1% significance test. Whether it is a rural or urban migrant individual, the impact of migration distance on happiness is always negative. However, the adverse effect of migration distance on the happiness of the urban migrant individuals is more robust than that of the rural ones, about 19.05% higher. Usually, the Chinese economy presents a clear urban-rural dual structure. The rural resident in China is poorer than the urban resident, which means that the pursuit of happiness is easier for the poor rural migrant individuals. These results in Table 5 verify Hypothesis 2.

**Table 5:** Heterogeneous effects of migration distance by household register.

**Variables**	**Rural**		**Urban**	
	**Coef**	**OR**	**Coef**	**OR**
Range	−0.357***	0.699***	−0.425***	0.653***
	(0.013)	(0.009)	(0.032)	(0.021)
Control variables	Yes		Yes	
LR chi^2^	1932.62		293.58	
Prob > chi^2^	0.0000		0.0000	
Pseudo R^2^	0.0105		0.0088	
Observations	86,122		15,287	

Standard errors in parentheses, ***p < 0.01.

### Analysis of Mediating Effect

The results of the mediating mechanism analysis of the impact of migration distance on happiness are shown in Table 6. The estimation results in column 2 indicate that the migration distance has a negative effect on social integration. Compared with intra-provincial migration, the effect of inter-provincial migration on social integration is −0.619, and both are significant at the 1% level. The social integration variable in column 3 is significant at the 1% level, with an estimated coefficient of 1.088, meaning that social integration has a significant positive effect on happiness.

**Table 6:** Mediating effect of social integration.

**Variable**	**Happiness**	**Social integration**	**Happiness**
	**Order logit**	**Logit**	**Order logit**
Range	−0.367***	−0.619***	−0.334***
	(0.012)	(0.028)	(0.012)
Social integration			1.088***
			(0.025)
Control variables	Yes	Yes	Yes
Constant		2.677***	
		(0.199)	
*N*	101,409	101,409	101,409

Standard errors in parentheses, ***p < 0.01.

From the above estimation results, it can be concluded that social integration plays a vital role in negative inhibition in the impact of migration distance on happiness. It means that the longer the migration distance, the weaker its social integration, resulting in a decline in the level of happiness. From the perspective of social integration, the migrant individuals who choose a close-range migration is more conducive to adapting to the new environment, overcoming differences in ideas, narrowing social exclusion, promoting healthy mental development, and continuously improving happiness. The results in Table 6 verify Hypothesis 3.

## Discussion

Happiness is the most direct reflection of the migrant individual's pursuit of welfare. Under the proximity trend of the migration distance in China, there is a complex causal relationship between migration distance and happiness.

This paper investigates the effect of migration distance on happiness by using the 2012 China Migrant Dynamic Survey. One of the most important findings to emerge from this paper is that the migration distance has a negative impact on happiness. PSM conducts further analysis on the endogeneity problem of migration distance on happiness. ATT based on k-nearest neighbor matching, kernel matching, local linear regression matching, and mahalanobis matching are all significantly negative, indicating that migration distance has a negative causal relationship with happiness. From the perspective of happiness, this paper explains the law of migration proposed by Ravenstein (43), which holds that the total number of migrants decreases with the extension of migration distance.

Heterogeneity analysis is conducted from the perspective of urban and rural migrant individuals. Our study discovers that urban individuals show a stronger migration distance response than rural counterparts. China's economy presents a prominent urban-rural dual structure, and the poor rural migrants' pursuit of happiness is easier to achieve.

In addition, our study also finds that social integration has a significant positive effect on happiness, which is consistent with previous findings on cultural integration on the happiness of the migrants (44–46). Social integration is shown to be the potential mechanism through which the effect of migration distance on happiness. This finding provides a valuable addition to the previous literature.

Our study comes with some limitations. Firstly, due to data constraints, we used the data from the 2012 China Migrants Dynamic Survey, which is a decade away from the present and may not reflect the happiness of the current migrant individuals. Secondly, the cross-sectional data used in this paper cannot reflect the effect of migration distance on happiness under temporal trends. Thirdly, this study only selected relevant control variables for personal characteristics. Existing studies have found that air pollution can not only affect happiness, but also affect individuals' migration decisions (47–50). Regretfully, due to the limitations of the questionnaire, the urban living environment, personal living conditions, and other real situations cannot be considered, which may cause deviations in the estimated results. Therefore, in future research, data and sample selection issues should be addressed to analyze better the causal effects of migration distance on happiness in a more realistic manner.

## Conclusion

In this study, we found that the migration distance of the Chinese internal migrants has a significant negative impact on happiness. From the perspective of urban-rural heterogeneity, urban individuals show a stronger response to migration distance compared to rural counterparts. Furthermore, we also find that social integration is an essential mechanism by which migration distance affects happiness.

Our findings highlight mental health issues such as the happiness of the migrant individuals and provide some practical implications in a targeted manner. First, it is necessary to respect the law of the migration distance. The government and society need to take appropriate measures to reduce discrimination in household registration and discriminatory laws and policies, pay attention to the equality of the mental health, education, pension, and other migrant issues, which help the migrants enjoy the equal public services as the original residents. Second, particular attention should be paid to social integration for happiness, such as strengthening cultural exchanges between different regions, promoting the social integration of traditional, and helping the migrants better integrate into the city. Third, the government should also focus more on coordinating regional economic development, narrowing the income gap between urban and rural areas, implement the lagging areas and their causes, and propose effective measures for the root causes of the problems to ensure that “Leave No One Behind.” Simultaneously, the government should promote rational migration of population and further improve relevant policies for the migrants.

## Data Availability Statement

The original contributions presented in the study are included in the article/supplementary material, further inquiries can be directed to the corresponding author.

## Author Contributions

GZ completed the research design, data analysis, writing the paper, and handled the revision of the manuscript. DY helped in developing the research idea, contributed some intellectual contents to the draft, and responsible for all R&R works. JL edited the manuscript. All authors contributed to the article and approved the submitted version.

## Funding

The authors acknowledge funding support from the Major Program Project of the National Social Science Fund of China (No: 21AJL006) and the Program Project Ministry of Education of China (No: 20JHQ079).

## Conflict of Interest

The authors declare that the research was conducted in the absence of any commercial or financial relationships that could be construed as a potential conflict of interest.

## Publisher's Note

All claims expressed in this article are solely those of the authors and do not necessarily represent those of their affiliated organizations, or those of the publisher, the editors and the reviewers. Any product that may be evaluated in this article, or claim that may be made by its manufacturer, is not guaranteed or endorsed by the publisher.
